# Ratio of Metastatic to Examined Lymph Nodes, a Helpful Staging System and Independent Prognostic Factor of Esophagogastric Junction Cancer

**DOI:** 10.1371/journal.pone.0073238

**Published:** 2013-08-19

**Authors:** Hao Zhang, Wei Wang, Dongmei Diao, Yao Cheng, Yongchun Song, Kun Zhu, Chengxue Dang

**Affiliations:** Oncology Department, the First Affiliated Hospital, Xi'an Jiaotong University, Xi’an, Shaanxi, China; University of Texas MD Anderson Cancer Center, United States of America

## Abstract

**Background:**

The incidence of the esophagogastric junction cancer is growing rapidly. The purpose of this study is to clarify the outcome of the ratio between metastatic and examined lymph nodes in esophagogastric junction cancer patients with or without 7 examined lymph nodes.

**Methods:**

A total of 3,481 patients who underwent operation are identified from the Surveillance, Epidemiology, and End Results database. Different lymph nodes resected groups are analyzed to test the lymph nodes ratio factor.

**Results:**

There are 2522 patients with 7 or more lymph nodes resected and 959 patients with less than 7 lymph nodes resected. Lymph nodes ratio and lymph node involvement are independent prognostic factors. But the lymph nodes ratio categories have a better prognostic value than the lymph node involvement categories. Compared with lymph node involvement categories, lymph nodes ratio categories represent patients with more homogeneous overall survival rate.

**Conclusions:**

This study defines that the lymph nodes ratio is an independent prognostic factor for esophagogastric junction cancer. The lymph nodes ratio can prevent stage migration and may be a helpful system to predict the prognosis of esophagogastric junction cancer patients.

## Introduction

The incidence of the esophagogastric junction cancer (EGJC) is growing rapidly in Western countries. But this trend has not occurred in Eastern countries [1–4]. According to the anatomic cardia, Siewert defines that EGJC can be divided into three subtypes: type I, adenocarcinoma of the distal esophagus with the center located within 1 cm above and 5 cm above the anatomic esophagogastric junction (EGJ); type II, true carcinoma of the cardia with the tumor center within 1 cm above and 2 cm below the EGJ; type III, subcardial carcinoma with the tumor center between 2 cm and 5 cm below EGJ, which infiltrates the EGJ and distal esophagus from below [5]. Just as incidence is different between Western and Eastern countries, the distribution of the three subtypes of EGJC differs between Eastern and Western countries [6]. In Eastern countries, type II and III cancers are more common than type I [7]. Even a retrospective study in Taiwan indicates no type I patient in the past 20 years [8]. But in Western countries, the distribution of the subtypes is nearly equal [9]. Consequently, based on the different characteristics, the treatment strategies, surgical technique and pathological analysis are diversed.

As a junctional cancer between esophagus and stomach, previous staging produced different stage groupings for these cancers depending on use of either esophageal or gastric stage system. The American Joint Committee on Cancer (AJCC) 7th edition and Union for International Cancer Control (UICC) staging system first harmonizes cancer staging across the esophagogastric junction [10]. But the optimal extent of lymph node dissection is still controversial. Compare with D1, D2 or D3 lymphadenectomy for gastric cancer or 3-field or 2-field lymphadenectomy for esophageal cancer, the extent of lymph nodes dissection of the EGJC is varied [11]. Meanwhile, more D1 lymphadenectomy are performed in Western countries than in Eastern countries that their patients generally receive more extensive lymphadenectomies [12,13]. The AJCC staging manual and some researches recommend 12 lymph nodes as the appropriate cut-off for lymphadenectomy in order to get a better survival benefit [14–16]. Therefore, it is important for an experienced pathologist to exam the specimen and for the surgeon to determine which technique modification is suitable for the lymphadenectomy [17].

Still some studies manifest that a number of patients do not receive a more than 12 lymph nodes lymphadenectomy. And this may result in a misleading of stage migration [18]. Lymph node ratio (LNR) is defined as the amount of positive lymph nodes divided by the amount of examined lymph nodes [19]. A number of researches propose LNR as a novel and independent prognostic factor that can reduce the phenomenon of stage migration in some other kinds of cancers [20,21]. So base on the fact that many population-based studies of esophagogastric or cardia cancer indicate that surgeons and pathologists failed to accomplish a satisfied lymphadenectomy, we choose LNR as a potential prognostic factor to reduce the stage migration, especially for the less than 7 examined lymph nodes group which cannot reach an exact pathological stage. This study analyzes approximately 3500 esophagogastric cancer patients from the Surveillance, Epidemiology, and End Results (SEER) database who underwent surgery between 1988 and 2009.

## Results

### Patients and the Demographic and Pathological Characteristics

There are 3481 patients in the SEER database who fulfilled the critical selection criteria between 1988 and 2009. Demographic and pathologic characteristics of group 1 and group 2 are summarized in Table 1 and Table 2 respectively.

**Table 1:** Univariate Analysis According to Clinicopathologic Factors in 2522 Patients with 7 or more Lymph Nodes Resected Who Underwent Surgery for AEG.

Factor	Patient (number)	5-years survival rate	95% CI	*P* value
All	2522	32		
Gender				0.261
Male	2039	31	61.6-79.4	
Female	483	35	62.7-71.2	
Age (years)				<0.001
<70	1627	34	73.4-84.1	
≥70	895	26	44.7-53.5	
Race				0.801
White	2215	31	63.3-71.5	
Black	102	34	46.3-79.1	
Others	202	38	57.1-81.2	
Unknown	3	33	9.4-29.9	
Tumor size (mm)				<0.001
<30	480	46	82.8-104.6	
≥30	1687	27	55.6-64.1	
Unknown	355	35	56.2-71.7	
Grade				<0.001
Well	128	51	90.4-129.0	
Moderately	830	37	66.3-79.7	
Poorly	1337	25	51.8-61.4	
Undifferentiated	79	25	33.9-62.3	
Unknown	148	49	88.9-130.2	
AJCC N stage				<0.001
N0	885	57	99.3-115.0	
N1	488	27	54.1-69.4	
N2	583	21	43.6-56.2	
N3	566	9	24.9-34.0	
Lymph node ratio				<0.001
LNR 0	885	57	99.3-115.0	
LNR 1	569	29	57.4-72.8	
LNR 2	471	20	41.9-54.9	
LNR 3	597	8	23.5-31.6	

**Table 2:** Univariate Analysis According to Clinicopathologic Factors in 959 Patients with Less Than 7 Lymph Nodes Resected Who Underwent Surgery for AEG.

Factor	Patient (number)	5-years survival rate	95% CI	*P* value
All	959	31		
Gender				0.195
Male	787	31	51.6-62.4	
Female	172	32	54.4-82.1	
Age (years)				<0.001
<70	570	34	61.5-76.5	
≥70	389	26	39.1-50.1	
Race				0.892
White	881	31	53.5-64.2	
Black	29	38	36.4-88.0	
Others	48	29	37.0-80.2	
Unknown	1	1	121.0-121.0	
Tumor size (mm)				<0.001
<30	275	43	65.1-85.9	
≥30	499	24	40.8-51.5	
Unknown	185	33	57.4-89.9	
Grade				<0.001
Well	51	41	47.3-83.5	
Moderately	303	35	56.7-75.8	
Poorly	478	25	42.9-54.8	
Undifferentiated	26	34	30.1-87.1	
Unknown	93	43	59.8-104.2	
AJCC N stage				<0.001
N0	529	45	73.2-89.4	
N1	283	16	28.9-39.6	
N2	147	11	21.2-34.3	
Lymph node ratio				<0.001
LNR 0	529	45	73.2-89.4	
LNR 1	59	27	34.1-65.4	
LNR 2	83	14	28.1-49.1	
LNR 3	288	11	22.5-31.0	

Most patients were male (81.2%) and white (88.9%). There are 2522 patients with 7 or more lymph nodes resected (group 1) and 959 patients with less than 7 lymph nodes resected (group 2). 44611 lymph nodes are examined in all patients. In group 1, patients who have 7 or more lymph nodes resection, there are a total of 41067 resected (median, 14; mean, 16.3; range, 7–78) among which 9928 lymph nodes (24.2%) are found to be metastatic. In group 2, a total of 3544 lymph nodes (median, 4; mean, 3.7, range, 1–6) are removed and examined, and 947 (26.7%) resulted metastatic.

All the prognostic factors are considered at univariate analysis. In group 1 and group 2 patients, the factors retained are the following: age, tumor size, grade, AJCC N stage and LNR. Multivariate analysis shows that both LNR and lymph node involvement are independent prognostic factors. But the LNR categories have a better prognostic value than the AJCC N categories for the reason that the LNR categories have a higher hazard ratio than the AJCC N categories (HR 1.450 versus 1.098). Meanwhile, if only combined LNR and AJCC N stage by using the Cox regress analysis, just the LNR indicates an independent prognostic factor.

### AJCC N and Lymph Node Ratio Categories

**Figure 1:**
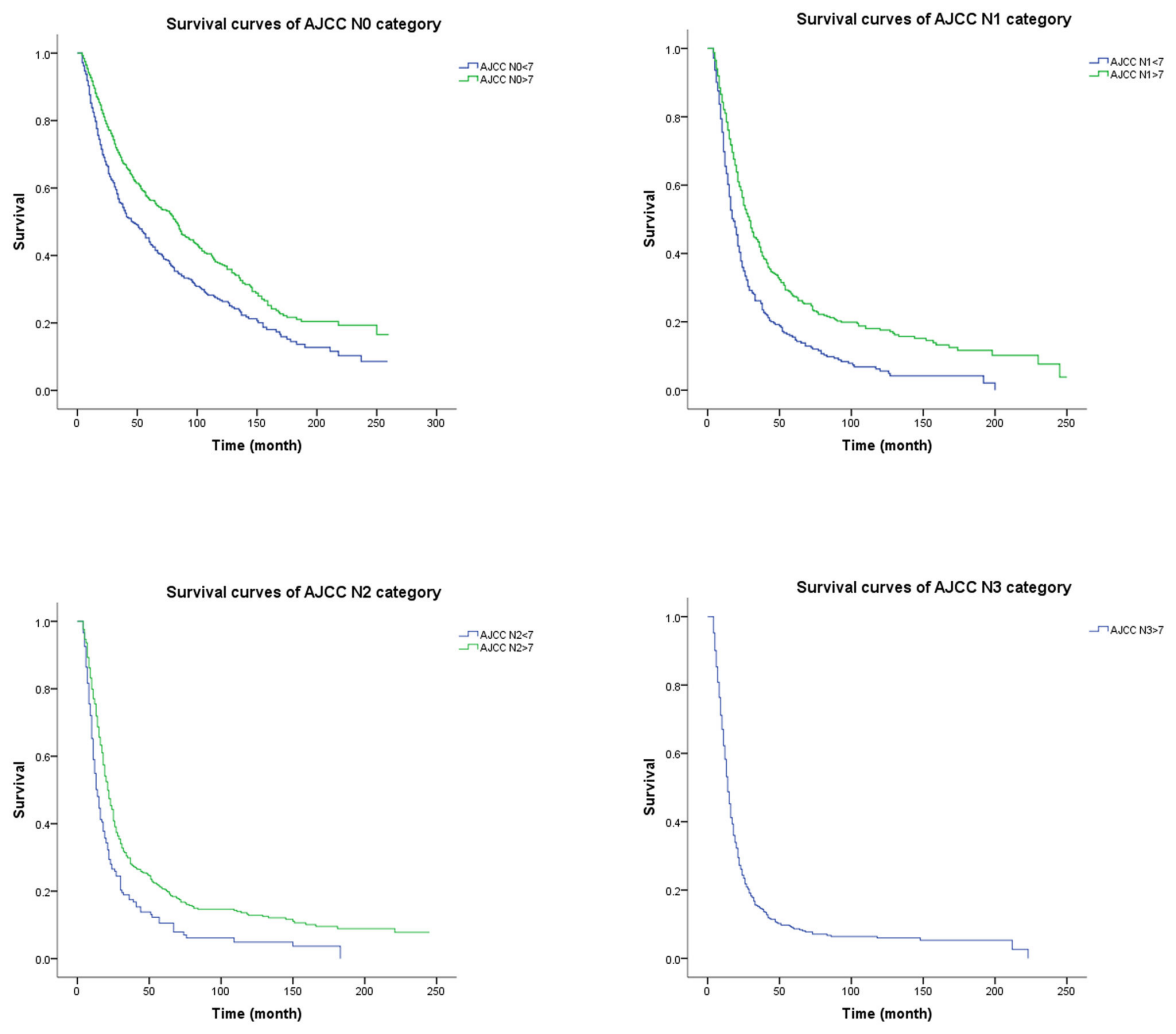
Overall survival according to AJCC N stage, stratified by the number of examined lymph nodes.

**Figure 2:**
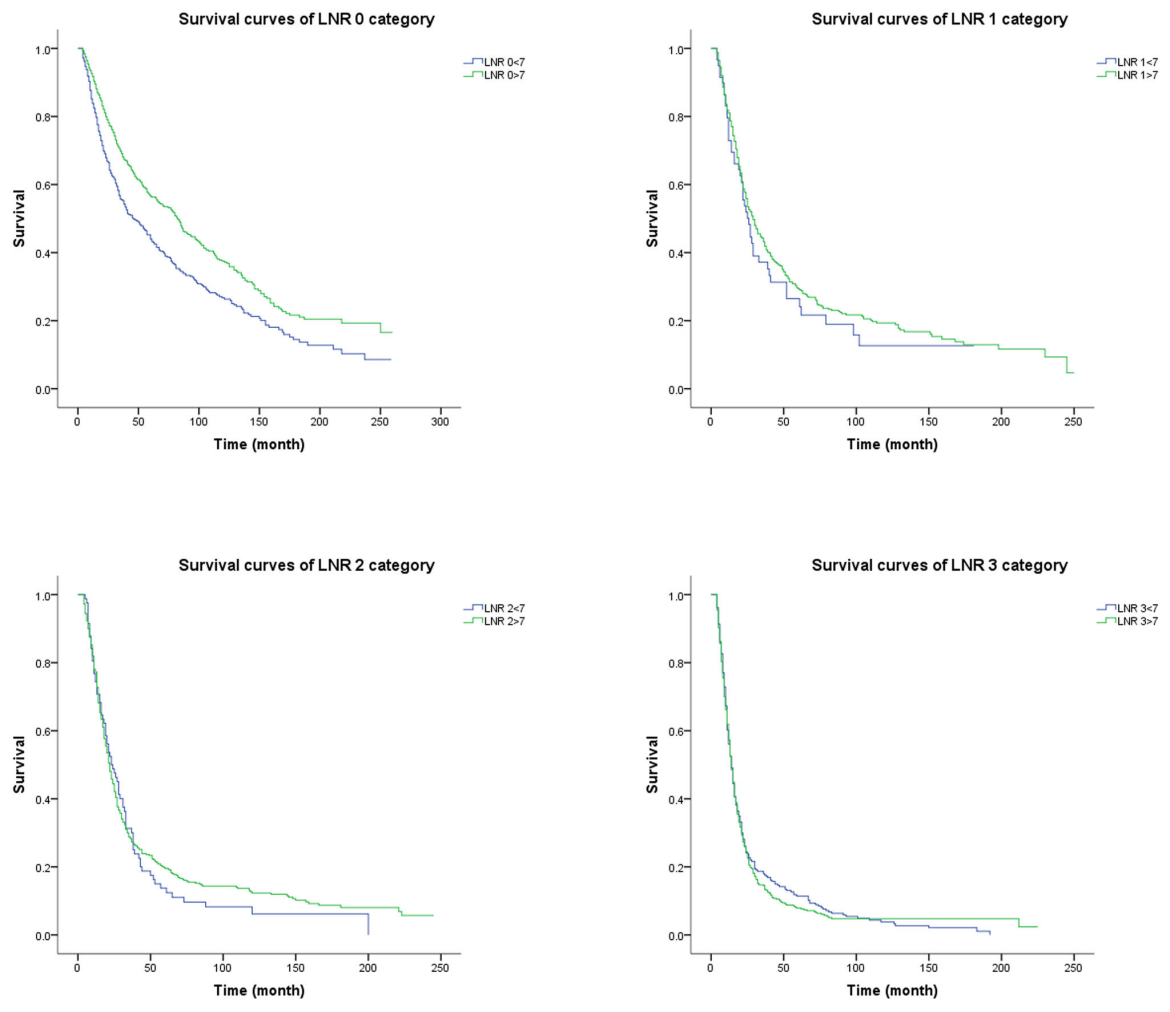
Overall survival according to LNR categories, stratified by the number of examined lymph nodes.

**Figure 3:**
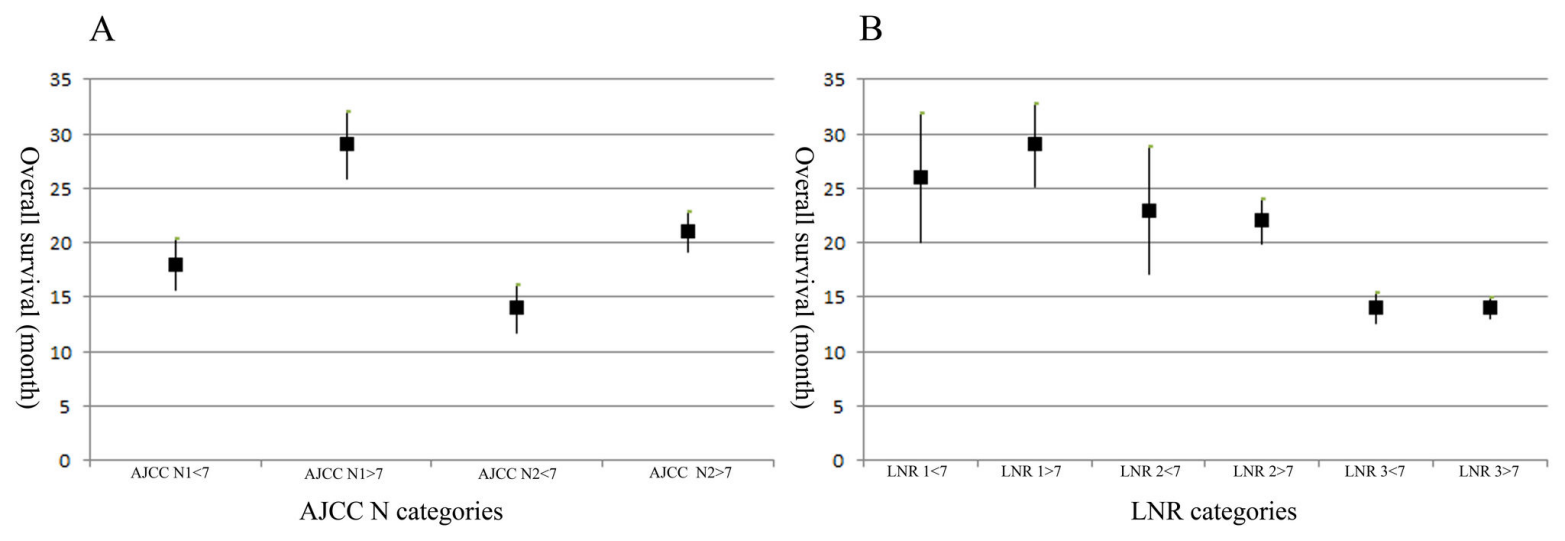
Comparison of prognostic discrimination power between the traditional AJCC N categories and LNR categories. Y axis, the mean and 95% confidence interval of overall survival (month). X axis, A. different AJCC N stage with 7 above lymph nodes resected or not; B. different LNR stage with 7 above lymph nodes resected or not.

For group 1 patients, the 5-year overall survival rate for the four AJCC N categories (N0-N3) is 57%, 27%, 21% and 9%, respectively (*P* < 0.001). Meanwhile, according to the LNR categories (LNR 0-LNR 3), the 5-year overall survival rate is 57%, 29%, 20% and 8%, respectively (*P* < 0.001). For group 2 patients, the situation is that the 5-year overall survival rate are 45%, 16% and 11% respectively (*P* < 0.001) in terms of the three AJCC N categories (N0-N2) and 45%, 27%, 14% and 11% respectively (*P* < 0.001) according the LNR categories (LNR 0-LNR 3). Compare the 5-year overall survival rate of the two subgroups, we can find that those with 7 or more lymph nodes examined have significantly better survival rate than those with less than 7 lymph nodes examined for all N categories (*P* < 0.001, Figure 1). But there is no significant difference in 5-year overall survival rate when the LNR 1, LNR 2, and LNR 3 categories are stratified into subgroups with 7 or more lymph nodes and less than 7 lymph nodes examined (Figure 2). What we need explain specially is that the AJCC N0 category and the LNR 0 category have same 5-year overall survival rate for the two subgroups because zero positive lymph node leads a zero numerator of LNR no matter how many lymph nodes are examined. This observation is also supported by the better prognostic discrimination associated with the LNR system when compared with that associated with the AJCC N system using the mean and 95% confidence interval (Figure 3). Thus, the 7th edition of AJCC N categories represent subgroups of patients with fairly wide ranges in overall survival when stratified by the number of examined lymph nodes. However, the use of LNR categories significantly reduces the range of overall survival within subgroups with 7 or more and less than 7 lymph nodes examined.

Additionally, we compare AJCC N stage and LNR categories. First, each N category (N0-N3) is stratified into LNR subgroups. After this stratification, each AJCC N stage is found to contain subgroups of patients with significantly heterogeneous 5-year overall survival rate (Table 3). The maximum difference in 5-year overall survival rate across subgroups is within the AJCC N3 category, where 5-year overall survival rate for the LNR 1 subgroup is 56% and for the LNR 3 subgroup 5%, representing a difference of 51%. Second, each LNR category (LNR 0-LNR 3) is stratified into AJCC N subgroups. After this stratification, the maximum difference in 5-year overall survival rate is within the LNR 1 category and this difference is only 28%. After scrutinize the data of the table, this super divergence is caused of the patients who are classified as AJCC N3 and LNR 1 which only contain 7 patients (unique subgroup less than 100 cases). However, if we remove the data which may result in the misleading, we can also find that the maximum difference is 20% within the AJCC N2 category and 8% within the LNR 3 category. These results indicate that compared with AJCC N categories, LNR categories represent patients with more homogeneous overall survival rate.

**Table 3:** 5-Year Overall Survival Rate Based on 7th Edition AJCC N Category and LNR Category.

	Lymph node ratio			
AJCC N stage	LNR 0	LNR 1	LNR 2	LNR 3
N0	52% (1414)^*^	-	-	-
N1	-	28% (496)	17% (134)	12% (141)
N2	-	33% (125)	18% (313)	13% (292)
N3	-	56% (7)	24% (107)	5% (452)

* 5-year overall survival rate (number of patients)

## Discussion

In this retrospective study, we investigated the prognostic value of LNR in a group of patients who underwent curative resection for EGJC from the SEER database. But the number of lymph nodes resected in 27.5% patients is less than 7 which cannot get an exact lymph nodes staging according to the 7th edition of the AJCC staging manual. Several potential reasons exist for this. First, EGJ, an anatomical junction which is not like the gastric cancer or esophageal cancer, is lack of detailed rules and regulations in searching the lymph nodes. Second, the number of lymph nodes resected is depended on the surgeon and the pathologist who varied in effort and technique as they searched for lymph nodes. Furthermore, as a junctional cancer, the surgery is done by the general surgeon or the thoracic surgeon and this may result in the confusion during the operation. Finally, the number of lymph nodes present in any given individual is variable and can be influenced by the patient, the tumor, and the treatment characteristics, such as the neoadjuvant chemotherapy [22,23].

The overall survival rate of group 1 is significantly different from that of group 2, both in AJCC N1 and N2 stage patients. We can conclude from the result that the number of metastatic lymph nodes may be underestimated if few lymph nodes are removed. And this can be reconfirmed by the AJCC N0 patients that those who have less than 7 lymph nodes resected have a significantly worse overall survival rate than those who have 7 or more lymph nodes resected. This finding may be due to the fact that some AJCC N0 patients from group 2 may turn out to be lymph nodes positive if a more extended lymphadenectomy have been performed. The underestimated AJCC N stage can lead to the understaging of patients which may limit the prognostic value of N stage. Further, this may result in some misleading of the treatment. But this phenomenon does not appear in LNR system. The overall survival rate of group 1 and group 2 does not show a significant difference in LNR 1 to LNR 3. The ratio between positive and examined lymph nodes has been proposed as a better category that can be used to identify subgroup of gastric and colon cancer patients [22,24–26]. The prevailing theory is that a thorough LNR evaluation results in more accurate staging and, thus, better determination of prognosis. But we need to know that in cancer staging systems, the staging recommendations apply to both clinical and pathologic staging. Clinical determination of positive lymph node number is possible and correlated with survival while we cannot measure the number of examined lymph nodes before the surgery. So the LNR is only measurable in pathologic staging.

According to the UICC/AJCC 7^th^ edition, A tumour, with the epicenter of which was within 5 cm of the esophagogastric junction (EGJ) and also extended into the esophagus, was classiﬁed and staged according to the esophageal scheme. And all other tumours with an epicenter in the stomach greater than 5 cm from the oesophagogastric junction or those within 5 cm of the esophagogastric junction without extension into the esophagus were staged using the gastric carcinoma scheme. The esophagus and stomach have different anatomical and histological structure. This may cause great controversy of the tumor staging, especially about the different depth of invasion. So LNR can be a helpful system to classify the EGJC. It is not just has a more homogeneous overall survival rate, it also can a better prognostic value than the AJCC stage categories for the reason that the LNR system has a higher hazard ratio than the AJCC stage categories (HR 1.541 versus 1.007) when only the two factors are compared in the Cox model (the data only include the patients who have exact AJCC stage in the SEER database).

In our study, the optimal cut-offs for lymph nodes ratios are determined using a cohort of patients with a median of 11 examined lymph nodes. The major advantage of this staging system is that it is applicable to the majority of patients undergoing operation with limited lymphadenectomy and it is already validated. But the cut-off value has not reached a explicit criterion. When we divide the LNR with 5% increment, we find that patients with LNR between 60% and 65% have an odd 5-year overall survival rate. They have a significantly better survival rate than the LNR between 45% and 60%. After carefully and furtherly analyse the data, we find that patients whose LNR are between 60% and 65% (35 patients with more than 7 lymph nodes examined) have more lymph nodes resected (median, 13; mean, 17.4, range, 8–59) than the other patients. Just as 7th AJCC manual, an appropriate lymphadenectomy may reach some prognostic values.

## Materials and Methods

### Patients

The SEER Program of the National Cancer Institute (NCI) is an authoritative source of information on cancer incidence and survival in the United States which covers approximately 28% of American population. Data collected include patient demographic information, pathological information and survival from 1988 to 2009. The Inclusion criteria are: 1. Patients with cancer located in esophagogastric junction; 2. ICD-O-3 code within the range of 8000–8152, 8154–8231, 8243–8245, 8247–8248, 8250–8576, 8940–8950, 8980–8981; 3. Patients who underwent surgery and exact pathological details can be achieved (include the amount of positive lymph nodes, the amount of examined lymph nodes and the depth of invasion); 4. Patients who lived more than three months after surgery; 5. Patients without distant metastasis. Then, the data from SEER are subdivided into two groups: Group 1 is composed of patients with 7 or more lymph nodes resected; Group 2 consists of patients with less than 7 lymph nodes resected. Because only patients who received the 7 above lymphadenectomy could make an accurate N stage according to the AJCC 7th edition.

### Statistical Analysis

Continuous data are presented as the mean ± standard deviation (SD). The survival status is analyzed by Kaplan-Meier survival curves, and univariate analysis is performed by using the log-rank test. Multivariate analysis is performed by using the Cox proportional hazards model. The three cut-off points are chosen by stratifying patients into 20 groups with 5% increment in node ratio. Then, three LNR stages are established by combining the neighborhood survival curves using the log-rank test [24]. The analyses identify the following best cut-off values: LNR 0, 0%; LNR 1, 0%–20%; LNR 2, 20%–45%; LNR 3, >45%. The following independent variables are analyzed: 1. Age (<70 years old versus ≥70 years old); 2. Sex (male versus female); 3. Race (white versus black versus others); 4. Tumor size (<30 mm versus ≥30 mm); 5. Grade (well versus moderately versus poorly versus undifferentiated); 6. AJCC N stage (N0 versus N1 versus N2 versus N3); 7. LNR (LNR 0 versus LNR 1 versus LNR 2 versus LNR 3). Statistical analyses were performed using SPSS 13.0. All statistical tests were conducted 2-sided, and *P* values < 0.05 were considered to be statistically signiﬁcant.

## Conclusions

In conclusion, this study defines that the LNR is an independent prognostic factor for esophagogastric junction cancer. The LNR can prevent stage migration and may be an helpful system to predict the prognosis of esophagogastric junction cancer patients.

## References

[1] Devesa SS, Blot WJ, Fraumeni JF (2000) Changing patterns in the incidence of esophageal and gastric carcinoma in the United States. Cancer 83: 2049-2053. PubMed: 9827707. 9827707

[2] Okabayashi T, Gotoda T, Kondo H, Inui T, Ono H et al. (2000) Early carcinoma of the gastric cardia in Japan. Cancer 89: 2555-2559. doi:10.1002/1097-0142(20001215)89:12. PubMed: 11135215. 11135215

[3] Blot WJ, Devesa SS, Kneller RW, Fraumeni JF Jr (1991) Rising Incidence of Adenocarcinoma of the Esophagus and nGastric Cardia. J Am Med Assoc 265: 1287-1289. doi:10.1001/jama.1991.03460100089030. 1995976

[4] Hasegawa S, Yoshikawa T (2010) Adenocarcinoma of the esophagogastric junction: incidence, characteristics, and treatment strategies. Gastric Cancer 13: 63-73. doi:10.1007/s10120-010-0555-2. PubMed: 20602191. 20602191 10.1007/s10120-010-0555-2

[5] Siewert J, Stein H (1996) Carcinoma of the gastroesophageal junction-Classification, pathology and extent of resection. Dis Esophagus 9: 173-182.

[6] Hasegawa S, Yoshikawa T, Cho H, Tsuburaya A, Kobayashi O (2009) Is adenocarcinoma of the esophagogastric junction different between Japan and western countries? The incidence and clinicopathological features at a Japanese high-volume cancer center. World J Surg 33: 95-103. doi:10.1007/s00268-008-9740-4. PubMed: 18958523. 18958523 10.1007/s00268-008-9740-4

[7] Hosokawa Y, Kinoshita T, Konishi M, Takahashi S, Gotohda N et al. (2012) Clinicopathological features and prognostic factors of adenocarcinoma of the esophagogastric junction according to Siewert classification: Experiences at a single institution in Japan. Ann Surg Oncol 19: 677-683. doi:10.1245/s10434-011-1983-x. PubMed: 21822549. 21822549 10.1245/s10434-011-1983-x

[8] Fang WL, Wu CW, Chen JH, Lo SS, Hsieh MC et al. (2009) Esophagogastric junction adenocarcinoma according to Siewert classification in Taiwan. Ann Surg Oncol 16: 3237-3244. doi:10.1245/s10434-009-0636-9. PubMed: 19636628. 19636628 10.1245/s10434-009-0636-9

[9] Siewert JR, Feith M, Werner M, Stein HJ (2000) Adenocarcinoma of the esophagogastric junction: results of surgical therapy based on anatomical/topographic classification in 1,002 consecutive patients. Ann Surg 232: 353–361. doi:10.1097/00000658-200009000-00007. PubMed: 10973385. 10973385 10.1097/00000658-200009000-00007 PMC1421149

[10] Rice TW, Blackstone EH, Rusch VW (2010) 7th Edition of the AJCC Cancer Staging Manual: esophagus and esophagogastric junction. Annals of surgical oncology 17: 1721-1724. 20369299 10.1245/s10434-010-1024-1

[11] Dresner SM, Lamb PJ, Bennett MK, Hayes N, Griffin SM (2001) The pattern of metastatic lymph node dissemination from adenocarcinoma of the esophagogastric junction. Surgery 129: 103-109. doi:10.1067/msy.2001.110024. PubMed: 11150040. 11150040 10.1067/msy.2001.110024

[12] Yoon SS, Yang HK (2009) Lymphadenectomy for gastric adenocarcinoma: should west meet east? Oncologist 14: 871-882. doi:10.1634/theoncologist.2009-0070. PubMed: 19738001. 19738001 10.1634/theoncologist.2009-0070

[13] Macdonald JS, Smalley SR, Benedetti J, Hundahl SA, Estes NC et al. (2001) Chemoradiotherapy after surgery compared with surgery alone for adenocarcinoma of the stomach or gastroesophageal junction. N Engl J Med 345: 725-730. doi:10.1056/NEJMoa010187. PubMed: 11547741. 11547741 10.1056/NEJMoa010187

[14] Edge SB, Compton CC (2010) The American Joint Committee on Cancer: the 7th edition of the AJCC cancer staging manual and the future of TNM. Annals of surgical oncology 17: 1471-1474. 20180029 10.1245/s10434-010-0985-4

[15] Groth SS, Virnig BA, Whitson BA, DeFor TE, Li ZZ et al. (2010) Determination of the minimum number of lymph nodes to examine to maximize survival in patients with esophageal carcinoma: data from the Surveillance Epidemiology and End Results database. J Thorac Cardiovasc Surg 139: 612-620. doi:10.1016/j.jtcvs.2009.07.017. PubMed: 19709685. 19709685 10.1016/j.jtcvs.2009.07.017

[16] Dutkowski P, Hommel G, Böttger T, Schlick T, Junginger T (2002) How many lymph nodes are needed for an accurate pN classification in esophageal cancer? Evidence for a new threshold value. Hepato Gastroenterol 49: 176. 11941947

[17] Herrera LJ (2010) Extent of lymphadenectomy in esophageal cancer: how many lymph nodes is enough? Ann Surg Oncol 17: 676-678. doi:10.1245/s10434-009-0824-7. PubMed: 19953331. 19953331 10.1245/s10434-009-0824-7 PMC2820691

[18] De Manzoni G, Verlato G, Roviello F, Morgagni P, Di Leo A, et al. (2002) The new TNM classification of lymph node metastasis minimises stage migration problems in gastric cancer patients. Br J Cancer 87: 171-174. doi:10.1038/sj.bjc.6600432. PubMed: 12107838. 12107838 10.1038/sj.bjc.6600432 PMC2376108

[19] Okusa T, Nakane Y, Boku T, Takada H, Yamamura M et al. (1990) Quantitative analysis of nodal involvement with respect to survival rate after curative gastrectomy for carcinoma. Surg Gynecol Obstet 170: 488–494. PubMed: 2343364. 2343364

[20] Bando E, Yonemura Y, Taniguchi K, Fushida S, Fujimura T et al. (2002) Outcome of ratio of lymph node metastasis in gastric carcinoma. Ann Surg Oncol 9: 775-784. doi:10.1007/BF02574500. PubMed: 12374661. 12374661 10.1007/BF02574500

[21] Inoue K, Nakane Y, Iiyama H, Sato M, Kanbara T et al. (2002) The superiority of ratio-based lymph node staging in gastric carcinoma. Ann Surg Oncol 9: 27-34. doi:10.1245/aso.2002.9.1.27. PubMed: 11829427. 11829427 10.1245/aso.2002.9.1.27

[22] Xu DZ, Geng QR, Long ZJ, Zhan YQ, Li W et al. (2009) Positive lymph node ratio is an independent prognostic factor in gastric cancer after d2 resection regardless of the examined number of lymph nodes. Ann Surg Oncol 16: 319-326. doi:10.1245/s10434-008-0240-4. PubMed: 19050970. 19050970 10.1245/s10434-008-0240-4

[23] Zhang ZX, Gu XZ, Yin WB, Huang GJ, Zhang DW et al. (1998) Randomized clinical trial on the combination of preoperative irradiation and surgery in the treatment of adenocarcinoma of gastric cardia (AGC)—report on 370 patients. Int J Radiat Oncol Biol Phys 42: 929-934. doi:10.1016/S0360-3016(98)00280-6. PubMed: 9869212. 9869212 10.1016/s0360-3016(98)00280-6

[24] Marchet A, Mocellin S, Ambrosi A, Morgagni P, Garcea D et al. (2007) The ratio between metastatic and examined lymph nodes (N ratio) is an independent prognostic factor in gastric cancer regardless of the type of lymphadenectomy: results from an Italian multicentric study in 1853 patients. Ann Surg 245: 543–552. doi:10.1097/01.sla.0000250423.43436.e1. PubMed: 17414602. 17414602 10.1097/01.sla.0000250423.43436.e1 PMC1877031

[25] Gao P, Song YX, Wang ZN, Xu YY, Tong LL et al. (2012) Integrated Ratio of Metastatic to Examined Lymph Nodes and Number of Metastatic Lymph Nodes into the AJCC Staging System for Colon Cancer. PLOS ONE 7: e35021. doi:10.1371/journal.pone.0035021. PubMed: 22529970. 22529970 10.1371/journal.pone.0035021 PMC3329536

[26] Berger AC, Sigurdson ER, LeVoyer T, Hanlon A, Mayer RJ et al. (2005) Colon cancer survival is associated with decreasing ratio of metastatic to examined lymph nodes. J Clin Oncol 23: 8706-8712. doi:10.1200/JCO.2005.02.8852. PubMed: 16314630. 16314630 10.1200/JCO.2005.02.8852

